# C-Peptide and BMi predict anti-hyperglycemic treatment lines in breast cancer patients treated with Alpelisib

**DOI:** 10.1007/s12020-024-03924-y

**Published:** 2024-07-04

**Authors:** Elena Carrillo-Lopez, Fernando Sebastian-Valles, Carolina Sager La Ganga, Anabel Ballesteros, Victor Navas-Moreno, Dulce Bañón, María Pilar López Martí, Mónica Marazuela, José Alfonso Arranz Martín

**Affiliations:** 1 Department of Endocrinology and Nutrition, Hospital Universitario de La Princesa, Instituto de Investigación Sanitaria de La Princesa. Universidad Autónoma de Madrid, 28006 Madrid, Spain; 2 Department of Oncology, Hospital Universitario de La Princesa, Instituto de Investigación Sanitaria de La Princesa, 28006 Madrid, Spain

**Keywords:** Alpelisib, breast cancer, glycated hemoglobin, body mass index, C-Peptide

## Abstract

**Purpose:**

Alpelisib is a PI3K (Phosphoinositide 3-kinases) inhibitor used for breast cancer which develops hyperglycemia based on its action on glucose metabolism regulation. This study aims to identify potential risk factors predicting hyperglycemia development and the need for multiple treatments for hyperglycemia in patients receiving Alpelisib.

**Methods:**

Fourteen women diagnosed with metastatic hormone receptor-positive breast cancer carrying PI3K mutations who initiated treatment with Alpelisib were monitored through consultations in the Oncology and Endocrinology departments. Non-parametric ROC curves were generated to assess the need for three or more antidiabetic medications to achieve glycemic control.

**Results:**

The study population had a median age of 64 years (range:48–69) with a median body mass index (BMI) of 26.6 kg/m^2^ (range: 22.9–29.4). Overweight was observed in 35.7% of the participants and obesity in 21.4%. Fifty percent of the participants had prediabetes, and 85.7% developed hyperglycemia requiring pharmacological treatment, although none of them needed to discontinue treatment for this reason. Baseline C-peptide levels and BMI were associated with the number of antidiabetic drugs used (Spearman’s Rho 0.553, p = 0.040; Spearman’s Rho 0.581, p = 0.030, respectively). ROC curve analysis showed and area under the curve (AUC) of 0.819 for the variable risk profile (defined as baseline C-peptide >10.5 ng/ml and BMI > 27 kg/m^2^), whereas AUC values were 0.556 and 0.514 for HbA1c and baseline glucose, respectively, (p = 0.012).

**Conclusion:**

A joint follow-up by an Oncology department and a Diabetes Unit can prevent treatment discontinuation in patients under Alpelisib therapy. Baseline BMI and plasma C-peptide levels can predict an increased need for anti-hyperglycemic treatment.

## Introduction

Breast cancer is the leading cause of cancer-related death in women worldwide [1, 2]. The most common subtype is characterized by hormone receptor-positive (HR+) and human epidermal growth factor receptor 2-negative (HER2-) tumors [3]. Accordingly, treatments targeting the blockade of these receptors are the primary therapeutic strategy for these tumors. Resistance to hormonal treatment has been associated with mutations in members of the phosphatidylinositol-3-kinase (PI3K) family, which are present in up to nearly 40% of breast cancers and correlate with worse prognosis [4].

Alpelisib is an oral selective inhibitor of the alpha subunit of PI3K indicated for postmenopausal women, and men, with advanced or metastatic HR + HER2- breast cancer harboring PI3K mutations. Although the use of Alpelisib increases progression-free survival in this patient population [5], the appearance of adverse events with this treatment remains one of the primary constraints to its utilization.

**Fig. 1:**
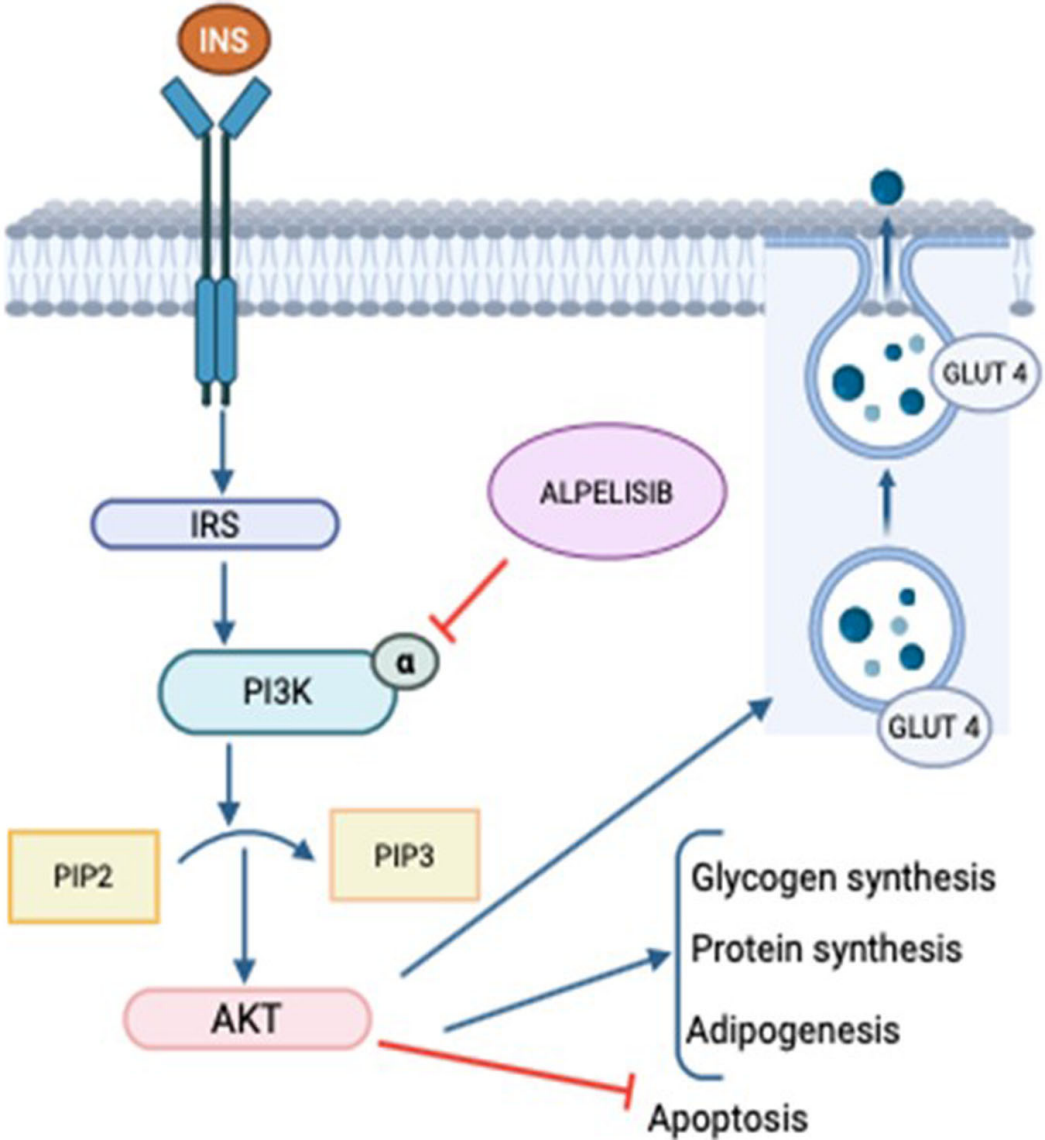
Alpelisib mechanism of action in insulin triggered PI3K signaling pathway. INS insulin, IRS insulin receptor, PI3K phosphatidylinositol-3-kinase, PIP2 phosphatidylinositol 4,5-bisphosphate PIP3: Phosphatidylinositol (3,4,5)-trisphosphate AKT: Protein Kinase B GLUT4: Glucose Transporter 4 Figure created with biorender

In addition to playing a role in cell proliferation pathways, PI3K plays an important role in intracellular signaling in glucose metabolism. Accordingly, PI3K inhibition by Alpelisib induces hyperglycemia and marked insulin resistance in more than 50% of patients treated with this drug; these conditions constitute the most frequent adverse event in Alpelisib treatment [5–8]. For this reason, poorly controlled type 1 (T1D) or type 2 (T2D) diabetes mellitus are considered contraindications for initiating treatment [9], which may present a limitation in clinical practice given the high prevalence of T2D in women with breast cancer who could be candidates for Alpelisib treatment [5, 7]. Alpelisib mechanism of action is described in Fig. 1.

Clinical practice guidelines for Alpelisib establish a series of risk factors for developing hyperglycemia and emphasize the importance of early initiation of anti-hyperglycemic treatment and collaborative glycemia monitoring with diabetes units to minimize its clinical impact [10]. Factors such as Asian ancestry, pre-existing hyperglycemia, baseline glycated hemoglobin (HbA1c), and high body mass index (BMI) are associated with an increased risk of Alpelisib-induced hyperglycemia [7]. Nevertheless, the effects of systematic follow-up of these patients in Diabetes Units have not been evaluated yet. Moreover, other metabolic factors associated with a risk profile for hyperglycemia development remain to be identified.

This study aimed to evaluate the efficacy of collaborative monitoring involving a Diabetes Unit and a Medical Oncology Department in controlling Alpelisib-induced hyperglycemia in real life, and to identify baseline risk factors capable of predicting the progression of this adverse event, and the need for multiple anti-hyperglycemic treatments.

## Methods

### Design

This is a retrospective cohort study conducted with adult women diagnosed with advanced or metastatic breast cancer who initiated Alpelisib treatment between March 2021 and July 2023 at a tertiary hospital in Madrid, Spain and were followed in the interdisciplinary oncology and endocrinology clinic.

The study enrolled patients with breast cancer harboring a PI3K mutation who started Alpelisib treatment at a dosage of 300 mg per day, combined with intramuscular Fulvestrant administered at a dose of 500 mg every 14 days the first month and every 28 days afterward. Patients with T1D and those with poorly controlled T2D, defined as an HbA1c level exceeding 7.5%, were excluded from the study.

### Procedures

All patients were referred from the Oncology Department to the Diabetes Unit at the initiation of oncological treatment with Alpelisib. All patients received a training session on diabetes management conducted by specialized nursing staff. Each patient was provided with a glucometer and received training for performing capillary glucose monitoring. Subsequently, all participants underwent comprehensive blood analysis including fasting basal glucose, HbA1c and C-peptide levels. Patients diagnosed with prediabetes (baseline glucose levels of 100–125 mg/dL or HbA1c levels of 5.7–6.5%) began treatment with metformin since the initial consultation. The dosage was progressively adjusted to promote gastrointestinal tolerance. For those with T2D, their antidiabetic treatment was optimized, adding additional medications at the endocrinologist’s discretion. Standardized joint visits by both services were conducted every 15 days until achieving glycemic control, defined as fasting glucose levels below 130 mg/dL. Pharmacological treatment was escalated sequentially based on fasting glucose monitoring, incorporating pioglitazone, a dipeptidyl peptidase 4 inhibitor (DPP4i), a sodium-glucose cotransporter-2 inhibitor (SGLT2i), basal insulin, and/or basal-bolus insulin regimen.

### Data collection

Data on sociodemographic and clinical characteristics, laboratory test results, and specific diabetes medications were retrieved from electronic medical records. Data included sex, age, breast cancer diagnosis date, Alpelisib initiation and discontinuation dates, Alpelisib dose reductions, reasons for discontinuation, baseline glucose levels, HbA1c, C-peptide, BMI, presence of hypertension, hyperlipidemia, molecular subtype of breast cancer, site and number of metastases. Glycated hemoglobin was routinely assessed using liquid chromatography (ADAMS A1c HA8180 V ARKRAY®).

All patients included in the study were informed about its objectives and provided consent for the utilization of data from their medical records for research purposes. The Research Ethics Committee of our Hospital, approved this study (Study Number: 2023-5347).

### Statistical analysis

Data were analyzed using STATA 17.0 BE-Basic Edition software (Lakeway Drive, College Station, TX, USA). Quantitative variables were described using medians and interquartile ranges (IQR: 25th–75th percentile), while qualitative variables were defined using frequencies and percentages. Normality of variables was assessed using the Shapiro-Wilk test. Subsequently, differences between groups were tested using the Mann-Whitney U test for non-normally distributed variables and the Student’s t-test for normally distributed variables. The chi-square test was employed for qualitative variables. Spearman’s rho test was used to evaluate correlations between variables.

The binary variable ‘need for intensive anti-hyperglycemic treatment’ was assessed, defined as the requirement for three or more antidiabetic medications or insulin. Non-parametric receiver operating characteristics (ROC) curves were generated to assess the diagnostic performance of various variables for the need for intensive anti-hyperglycemic treatment and were compared with reference parameters such as HbA1c or baseline glucose levels. A ‘risk profile’ variable was constructed using cutoff points from ROC curves of variables statistically associated with the need for intensive anti-hyperglycemic treatment. Multivariable linear regression models were executed to assess the strength of the risk profile to predict the need for intensive anti-hyperglycemic treatment Finally, the impact of the risk profile on Alpelisib drug discontinuation and mortality was investigated using Kaplan-Meier analysis and Log-rank statistics. Statistical significance was considered at p < 0.05.

## Results

### Patient characteristics

Fourteen women diagnosed with advanced HR+/HER2– breast cancer carrying a PIK3CA mutation who initiated therapy with Alpelisib were included in the analysis. Demographic characteristics of the patient cohort are detailed in Table 1. The median age was 64 years (range 48–69), and 100% of the patients were of Caucasian ethnicity. The most common weight phenotype was normal weight (42.9%), although 35.7% of patients were overweight, and 21.4% were obese. All patients were postmenopausal. One patient (7.1%) had a history of T2D, and 7 (50%) met prediabetes criteria based on fasting glucose and/or HbA1c at the initiation of Alpelisib. Seven participants (50%) had first-degree family history of T2D. The median baseline glucose level in the cohort at the study’s onset was 88 mg/dL (range 85 to 94), and the median baseline HbA1c was 5.7% (range 5.4 to 5.9). Peptide-C levels recorded at the initiation of Alpelisib treatment were 9.9 ng/mL (3.27 nmol/L) (range 6.9–11.5 ng/mL).

**Table 1:** Baseline characteristics of the study population

Variable	Obs *n* = 14
Sex (women), n (%)	14 (100%)
Age (years)	64.4 (48.2–69.6)
Ethnicity, n (%)	
Caucasian	14 (100%)
Hypertension, n (%)	3 (21.4%)
Hyperlipidemia, n (%)	5 (35.7%)
Diabetes history n (%)	0 (0%)
Gestational diabetes	7 (50%)
Prediabetes	1 (7.1%)
Type 2 Diabetes	6 (42.9%)
None	7 (50.0%)
First-degree relatives with diabetes mellitus	
HbA1c baseline (%)	5.7 (5.4–5.9)
HbA1c end follow-up(%)	6.5 (6–6.6)
Basal glucose (mg/dL)	88 (85–94)
C – Peptide (ng/dL)	9.9 (6.9–11.5)
BMI (Kg/m^2^)	26.6 (22.9–29.4)
Normal (18.6–24.9)	6 (42.9%)
Overweight (25–29.9)	5 (35.7%)
Obese (≥30)	3 (21.4%)
Cardiovascular disease (EF < 50%, arrhythmia)	0 (0%)

Quantitative data are expressed as median and p25-p75 range

*BMI* body mass Index, *HbA1c* Glycated hemoglobin, *EF* ejection fraction

Breast cancer characteristics and oncological treatment details are summarized in Table 2. At the time of data collection, two patients (14.3%) were still undergoing therapy with Alpelisib. The median treatment duration among those who discontinued treatment was 14 months (range 4.3–23.9). The most common reason for Alpelisib discontinuation was disease progression [9 patients (75%)]. Only one patient ceased treatment due to a skin rash, and two patients discontinued Alpesilib for other reasons (mucositis and an allergic reaction). None of the patients discontinued treatment due to hyperglycemia.

**Table 2:** Oncologic clinical characteristics

Variable	Obs *n* = 14
Alpelisib dose at initiation, n (%)	
200 mg/dl	1 (7.1%)
300 mg/dl	13 (92.9%)
Alpelisib dose decrease required, n (%)	10 (71.4%)
Median duration of alpelisib, months (range)	14 (4.3–23.9)
Reason for alpelisib discontinuation, n (%)	12 (85.7%)
Disease progression	9 (75%)
Rash	1 (8.3%)
Hyperglycemia	0 (0%)
Other	2 (16.7%)
Time from diagnosis to initiation of alpelisib (years)	10.3 (5.3–16.1)
Molecular Subtype of Breast Cancer, n (%)	
Luminal A	4 (28.6%)
Luminal B	2 (14.3%)
Unspecified Luminal	4 (28.6%)
Unknown	4 (28.6%)
Metastasis	
Bone	11 (78.6)
Liver	5 (35.7%)
Lung	5 (35.7)
Other	5 (35.7%)
Number of metastatic sites, n (%)	
1	7 (50%)
2	1 (7.1%)
3	4 (28.6%)
4	2 (14.3%)
CA 15–3 U/mL	160 (69–394)
CEA ng/mL	9.27 (4.32–63)

Data are expressed as median and p25-p75 range

*CA15-3* cancer antigen 15-3, *CEA* carcinoembryonic antigen

### Alpelisib-induced hyperglycemia

Twelve patients (85.7%) experienced hyperglycemia requiring the initiation of antidiabetic therapy. Of them, three patients (21.4%) achieved glycemic control with metformin, while five patients (35.7%) required metformin in combination with pioglitazone. One patient (7.1%) needed three medications, and three patients (21.4%) required insulin in addition to oral medications. The median HbA1c level at at the end of the study period for patients continuing the medication or at the time of Alpelisib discontinuation was 6.5% (range 6–6.6).

As the patients were under Diabetes Unit follow-up since the initiation of treatment, therapeutic adjustment and optimization were promptly performed when needed, preventing severe hyperglycemia events requiring Alpelisib interruption or discontinuation. The severity of Alpelisib-induced hyperglycemia was assessed through the number of different types of anti-hyperglycemic drugs required for diabetes control.

**Fig. 2:**
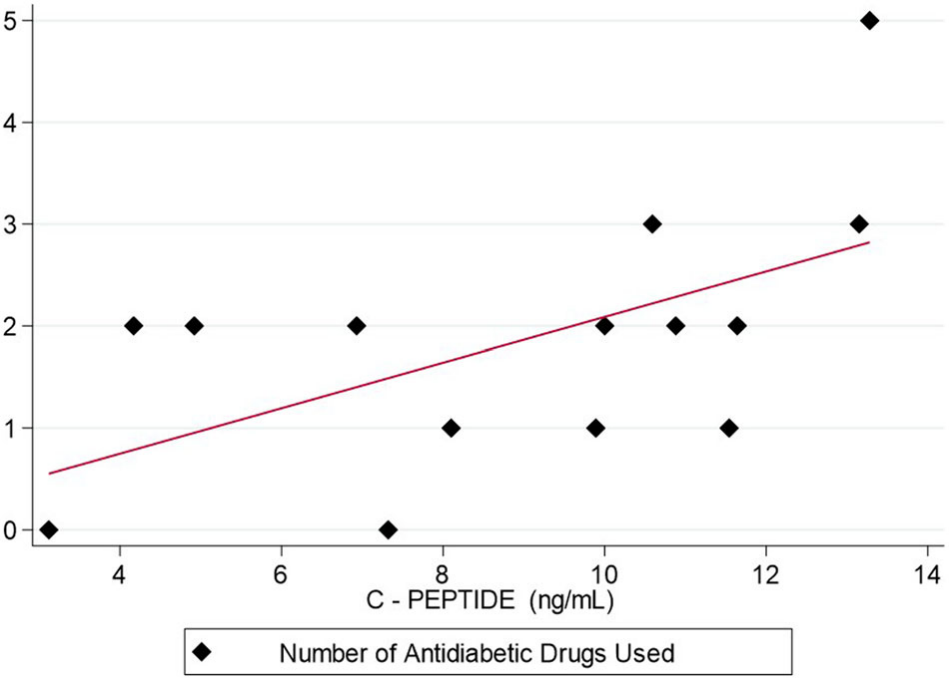
Association between baseline C-peptide and the number of different antidiabetic medications used for glycemic control. A statistically significant association was observed between basal C-peptide titers and the number of different antidiabetic drugs required to control Alpelisib-induced hyperglycemia (Rho Spearman 0.553 p = 0.040)

A positive association was observed between C-Peptide or BMI levels and the number of different antidiabetic medications used for glycemic control (Spearman’s Rho 0.553, p = 0.040; Spearman’s Rho 0.581, p = 0.030, respectively, Fig. 2). C-peptide and BMI were also associated (Spearman’s Rho 0.662, p = 0.010). Baseline glucose and HbA1c levels at the start of treatment did not correlate with the number of medications used (p = 0.820, p = 0.278, respectively).

**Fig. 3:**
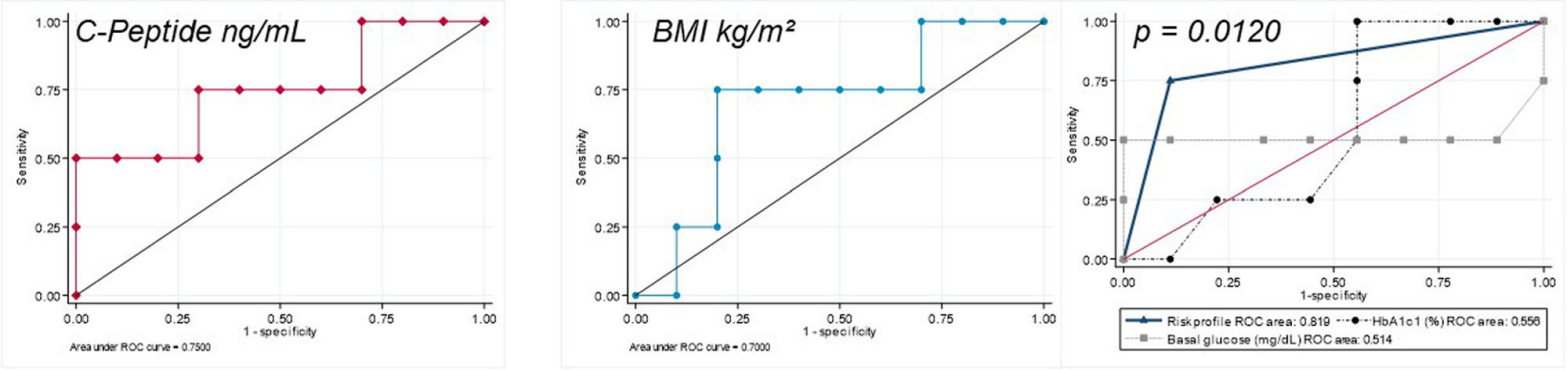
ROC curves analysis of C-Peptide, BMI and risk profile, glycosylated hemoglobin, and baseline glucose for the need for intensive antihyperglycemic treatment. Statistically significant differences were observed between the predictive power of risk profile (C-Peptide >10.5 ng/mL and BMI > 27 kg/m2) (AUC = 0.819) versus glycated hemoglobin (AUC = 0.556) and basal glucose (AUC = 0.514), p = 0.012

The predictive capacity of variables significantly associated with the need for intensive antihyperglycemic treatment to control Alpelisib-induced hyperglycemia was assessed using ROC curves. The area under the curve (AUC) for C-peptide was 0.750. The ROC curve cutoff point for C-peptide was determined as 10.5 ng/mL, with a sensitivity of 75% and a specificity of 70%. AUC for BMI at treatment onset was 0.700. The ROC curve cutoff point for BMI at treatment onset was 27 kg/m2, with a sensitivity of 75% and specificity of 70% (Fig. 3).

Following these results, a variable termed ‘risk profile’ was created, encompassing patients with a baseline C-peptide >10.5 ng/mL and an initial BMI > 27 mg/dL. To compare the capacity to predict intensive antihyperglicemic treatment of the risk phenotype with other classical variables used to study Alpelisib-induced hyperglycemia. New ROC curves were constructed for baseline glucose and HbA1c and compared with those of the risk profile using non-parametric methods. In this analysis the AUC for risk profile was 0.819, whereas AUCs for HbA1c and baseline glucose were 0.556 and 0.514, respectively, (p = 0.012) (Fig. 3).

**Fig. 4:**
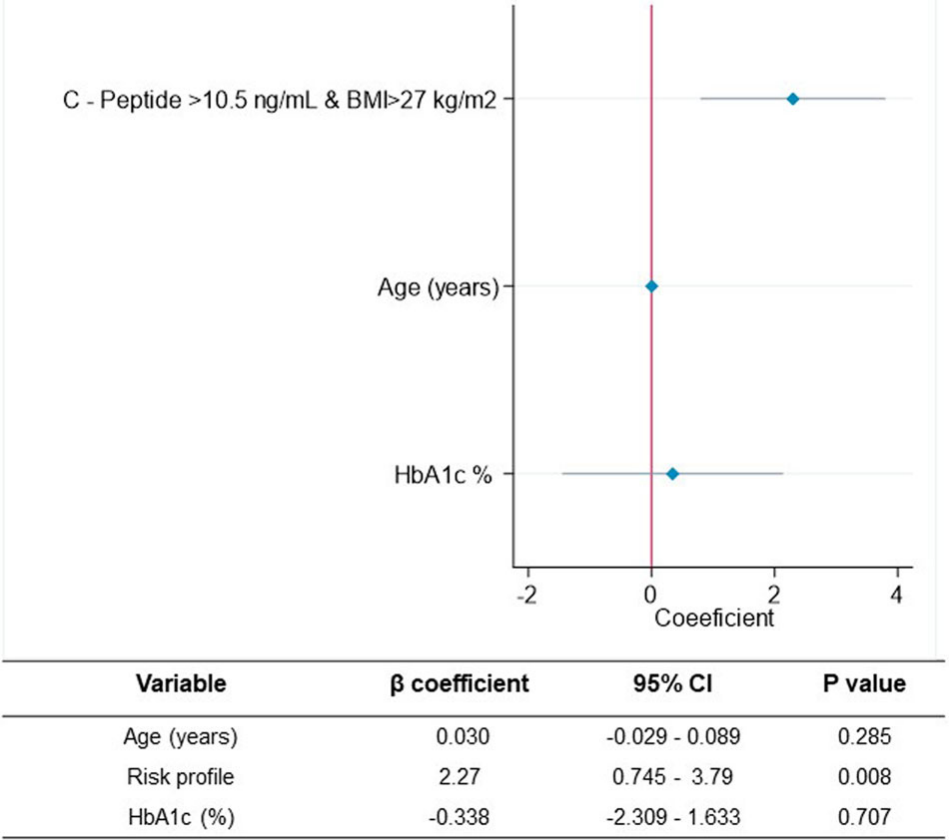
Predictors associated with an increased need for antidiabetic therapeutic lines. A statistically significant association was observed between risk profile (C-Peptide >10.5 ng/mL and BMI > 27 kg/m2) and the number of antidiabetic drug lines required independent of age and HbA1c, β = 2.27 (95% CI 0.745–3.79, p = 0.008)

In order to quantify the impact of the risk profile on the need for different pharmacological approaches to manage Alpelisib-induced hyperglycemia, a multivariable linear regression model adjusted for age and HbA1c was constructed. This analysis revealed a statistically significant association between the risk profile [β = 2.27 (95% CI 0.745–3.79, p = 0.008)] and the need for a higher number of antidiabetic pharmacological interventions, independent of age and HbA1c (p > 0.05) (Fig. 4).

**Fig. 5:**
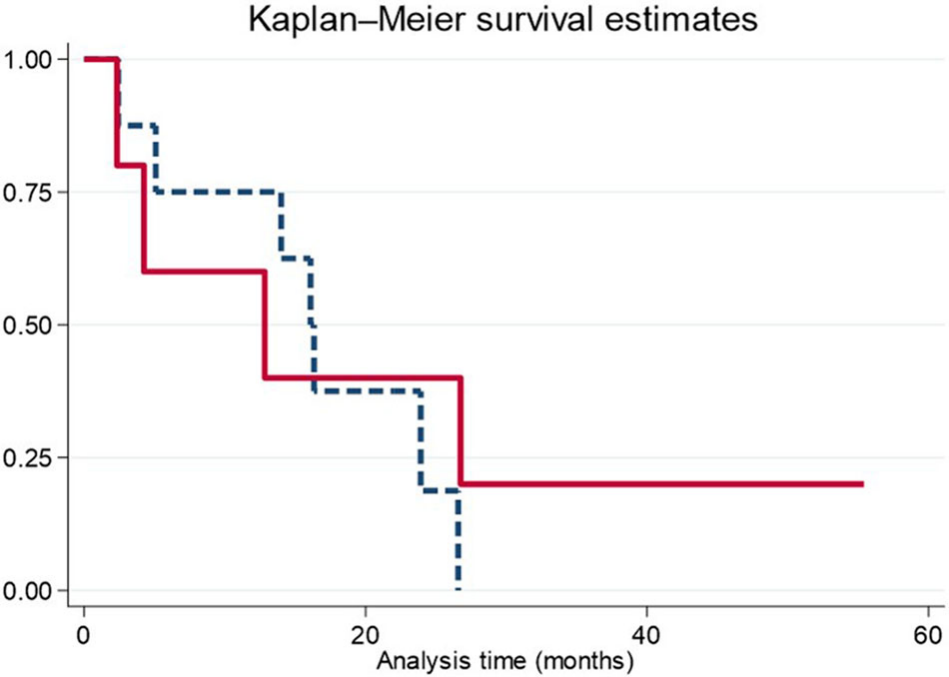
Survival model estimating the association between the risk profile and discontinuation of Alpelisib treatment. No statistically significant differences were observed in the discontinuation of Alpelisib treatment between the presence and absence of the risk profile (C-Peptide >10.5 ng/mL and BMI > 27 kg/m^2^)

Lastly, the study examined whether the risk profile was associated with an early discontinuation of Alpelisib treatment using a Kaplan-Meier survival model. No statistically significant differences were observed between patients who exhibited the risk profile and those who did not (log Rank = 0.614) (Fig. 5). Additionally, no association was found between the risk profile and mortality (log Rank = 0.872).

## Discussion

Alpelisib-induced hyperglycemia represents the most prevalent adverse event associated with treatment with this drug, occasionally posing a challenge to treatment continuity. Our study demonstrates that a collaborative intervention between a Diabetes Unit and an Oncology Department enables effective glycemic control during follow-up, thereby averting the need for treatment discontinuation due to this cause. Furthermore, our findings identify C-peptide and BMI as factors associated with an increased need for anti-hyperglycemic pharmacological interventions.

Some analysis of pivotal studies underscored the significance of pre-treatment glucose, HbA1c levels [11], BMI age and monocytes in the incidence of hyperglycemia as an adverse event of Alpelisib treatment [12]. Consequently, the need for increased glycemic monitoring to mitigate the clinical impact of hyperglycemia and the discontinuation of oncological treatment has been recognized [6]. Therefore, implementing protocols and seeking guidance from a diabetologist has been recommended for patients scheduled for Alpelisib treatment [10, 13].

Along these lines, the joint follow-up between the Diabetes Unit and the Oncology Department of our center resulted in a lower incidence of moderate or severe hyperglycemia (grades 3 and 4) and consequently a reduced incidence of dose reduction, temporary suspension, or definitive discontinuation of Alpelisib compared to other studies [5, 11, 13]. These favorable outcomes might be attributed to early training sessions on management of hyperglycemia at treatment onset and closer glycemic monitoring in these patients, as previously described in other reports [13].

Several sociodemographic, clinical, or laboratory parameters such as age, BMI, fasting plasma glucose, and HbA1c have been identified as factors capable of predicting the onset and severity of hyperglycemia in patients treated with PI3K inhibitors [12, 14]. In our series, fasting glucose and HbA1c before treatment with Alpelisib did not exhibit sufficient predictive power. This discrepancy might be explained by the absence of carbohydrate metabolism alterations in the majority of our patients and the different characteristics of the study populations.

Insulin and C-peptide are co-secreted by the pancreas in an equimolar ratio. Unlike insulin, C-peptide undergoes minimal hepatic degradation, and its plasma concentration follows a linear kinetics. Therefore, plasma C-peptide levels have been suggested to be more suitable than plasma insulin levels to monitor pancreatic insulin secretion. Consequently, C-peptide determination is valuable to differentiate between T1D and T2D [15]; moreover, C-peptide levels are associated with the need for insulin therapy in T2D [16]. Elevated C-peptide levels are also linked to insulin resistance and BMI [17]; furthermore, there is speculation regarding the potential independent role of C-peptide in atherosclerosis initiation [18]. However, C-peptide involvement in Alpelisib-induced hyperglycemia had not been explored before the current study. Our study establishes a risk profile based on C-peptide and BMI baseline levels. Defining a risk phenotype may allow planning more intensive diabetes monitoring strategies aimed at high-risk patients, such as continuous glucose monitoring. This system has been useful to understand Alpelisib pharmacodynamics and has been a valuable tool for managing and controlling hyperglycemia [19]. Predictive tools like the risk profile we propose could aid in identifying patients more likely to develop significant hyperglycemia, thereby facilitating the selective prescription of glucose-lowering medications in cases where it is truly necessary.

This study has several limitations. Firstly, its retrospective design limits the ability to establish causality, only allowing to merely propose hypotheses. The absence of a control group complicates the thorough verification of the intervention impact on patient follow-up. Additionally, the majority of patients in the study discontinued treatment due to disease progression, thus hampering medium-term Alpelisib follow-up. In addition, our study is limited to a small population from a single hospital. These limits cannot guarantee the generalizability of the results obtained in the present study, Thus, no general messages can be drawn but only preliminary data or suggestions. Therefore, further studies with larger and more diverse cohorts should be conducted before the risk factors identified in our study could be extrapolated to other populations, especially to populations of Asian descent, where the impact of BMI and insulin resistance differs from that of populations of Caucasian descent [7, 20].

In summary, hyperglycemia is a highly prevalent adverse event in women with metastatic breast cancer treated with Alpelisib. Follow-up coordinated by both Diabetes and Oncology Units can effectively manage glycemic levels and therefore prevent drug discontinuation due to this cause. Baseline C-peptide and BMI at the onset of treatment can be associated with an increased need for anti-hyperglycemic treatment. Given the methodological limitations of this study, future prospective multicenter studies should be conducted to verify the hypotheses proposed here.

## Data Availability

The datasets used and/or analyzed during the current study are available from the corresponding author on reasonable request.

## References

[1] Cancer Today n.d. https://gco.iarc.fr/today/home (accessed November 23, 2023)

[2] International Variation in Female Breast Cancer Incidence and Mortality Rates | Cancer Epidemiology, Biomarkers & Prevention | American Association for Cancer Research n.d. https://aacrjournals.org/cebp/article/24/10/1495/70670/International-Variation-in-Female-Breast-Cancer (accessed November 23, 2023) 10.1158/1055-9965.EPI-15-0535 26359465

[3] N. Howlader, S.F. Altekruse, C.I. Li, V.W. Chen, C.A. Clarke, L.A.G. Ries et al. US incidence of breast cancer subtypes defined by joint hormone receptor and HER2 Status. JNCI J. Natl. Cancer Inst. **106**, dju055 (2014). 10.1093/jnci/dju055 24777111 10.1093/jnci/dju055 PMC4580552

[4] E.J. Anderson, L.E. Mollon, J.L. Dean, T.L. Warholak, A. Aizer, E.A. Platt et al. A systematic review of the prevalence and diagnostic workup of PIK3CA mutations in HR+/HER2– metastatic breast cancer. Int. J. Breast Cancer **2020**, 3759179 (2020). 10.1155/2020/3759179 32637176 10.1155/2020/3759179 PMC7322582

[5] F. André, E. Ciruelos, G. Rubovszky, M. Campone, S. Loibl, H.S. Rugo et al. Alpelisib for *PIK3CA* -mutated, hormone receptor–positive advanced breast cancer. N. Engl. J. Med **380**, 1929–1940 (2019). 10.1056/NEJMoa1813904 31091374 10.1056/NEJMoa1813904

[6] H.S. Rugo, F. Lerebours, E. Ciruelos, P. Drullinsky, M. Ruiz-Borrego, P. Neven et al. Alpelisib plus fulvestrant in PIK3CA-mutated, hormone receptor-positive advanced breast cancer after a CDK4/6 inhibitor (BYLieve): one cohort of a phase 2, multicentre, open-label, non-comparative study. Lancet Oncol. **22**, 489–498 (2021). 10.1016/S1470-2045(21)00034-6 33794206 10.1016/S1470-2045(21)00034-6

[7] X. Ge, C.E. Behrendt, S.E. Yost, N. Patel, R. Samoa, D. Stewart et al. Predicting hyperglycemia among patients receiving alpelisib plus fulvestrant for metastatic breast cancer. Oncologist **28**, e488–e492 (2023). 10.1093/oncolo/oyad024 36943382 10.1093/oncolo/oyad024 PMC10322119

[8] S. Shen, Y. Chen, A. Carpio, C. Chang, N.M Iyengar. Incidence, risk factors, and management of alpelisib-associated hyperglycemia in metastatic breast cancer. Cancer (2023). 10.1002/cncr.34928 10.1002/cncr.34928 PMC10863751 37743730

[9] N.A. ElSayed, G. Aleppo, V.R. Aroda, R.R. Bannuru, F.M. Brown, D. Bruemmer et al. 2. classification and diagnosis of diabetes: standards of care in diabetes—2023. Diab. Care **46**, S19–S40 (2023). 10.2337/dc23-S002 10.2337/dc23-S002 PMC9810477 36507649

[10] E.J. Gallagher, H. Moore, M.E. Lacouture, S.F. Dent, A. Farooki, M.D. Goncalves et al. Managing hyperglycemia and rash associated with alpelisib: expert consensus recommendations using the Delphi technique. NPJ Breast Cancer **10**, 12 (2024). 10.1038/s41523-024-00613-x 38297009 10.1038/s41523-024-00613-x PMC10831089

[11] H.S. Rugo, F. André, T. Yamashita, H. Cerda, I. Toledano, S.M. Stemmer et al. Time course and management of key adverse events during the randomized phase III SOLAR-1 study of PI3K inhibitor alpelisib plus fulvestrant in patients with HR-positive advanced breast cancer. Ann. Oncol. J. Eur. Soc. Med. Oncol. **31**, 1001–1010 (2020). 10.1016/j.annonc.2020.05.001 10.1016/j.annonc.2020.05.001 32416251

[12] J. Rodón, D. Demanse, H.S. Rugo, H.A. Burris, R. Simó, A. Farooki et al. A risk analysis of alpelisib-induced hyperglycemia in patients with advanced solid tumors and breast cancer. Breast Cancer Res BCR **26**, 36 (2024). 10.1186/s13058-024-01773-1 38439079 10.1186/s13058-024-01773-1 PMC10913434

[13] S.E. Burnette, E. Poehlein, H.-J. Lee, J. Force, K. Westbrook, H.N. Moore, Evaluation of alpelisib-induced hyperglycemia prophylaxis and associated risk factors in PIK3CA-mutated hormone-receptor positive, human epidermal growth factor-2 negative advanced breast cancer. Breast Cancer Res. Treat. **197**, 369–376 (2023). 10.1007/s10549-022-06798-8 36409396 10.1007/s10549-022-06798-8

[14] G. Kim, M. Yoo, M.H. Hong, B.-W. Lee, E.S. Kang, B.-S. Cha et al. Predictive factors for the development of diabetes in cancer patients treated with phosphatidylinositol 3-kinase inhibitors. Cancer Chemother. Pharm. **84**, 405–414 (2019). 10.1007/s00280-019-03889-0 10.1007/s00280-019-03889-0 31250153

[15] A. Pujia, C. Gazzaruso, T. Montalcini, An update on the potential role of C-peptide in diabetes and osteoporosis. Endocrine **58**, 408–412 (2017). 10.1007/s12020-017-1286-5 28374151 10.1007/s12020-017-1286-5

[16] R. Uehara, E. Yamada, Y. Nakajima, A. Osaki, S. Okada, M. Yamada, Casual C peptide index: Predicting the subsequent need for insulin therapy in outpatients with type 2 diabetes under primary care. J. Diab. **14**, 221–227 (2022). 10.1111/1753-0407.13257 10.1111/1753-0407.13257 PMC9060140 35229479

[17] V. Kron, M. Verner, P. Smetana, J. Janoutova, V. Janout, K. Martinik, Alterations of glycaemia, insulin resistance and body mass index within the C-peptide optimal range in non-diabetic patients. J. Appl Biomed. **18**, 136–142 (2020) 34907766 10.32725/jab.2020.018

[18] D. Bruemmer, C-peptide in insulin resistance and vascular complications. Circ. Res. **99**, 1149–1151 (2006). 10.1161/01.RES.0000251785.83860.3b 17122442 10.1161/01.RES.0000251785.83860.3b PMC1829170

[19] B. Pla Peris, A. Arranz Martin, A. Ballesteros García, F. Sebastián-Valles, M Marazuela Azpiroz. Alpelisib-induced diabetes mellitus: case report, pharmacodynamics and management considerations. Front Endocrinol (2022);13. 10.3389/fendo.2022.802612 10.3389/fendo.2022.802612 PMC8843667 35178031

[20] T. Ohkura, H. Shiochi, Y. Fujioka, K. Sumi, N. Yamamoto, K. Matsuzawa et al. 20/(fasting C-peptide × fasting plasma glucose) is a simple and effective index of insulin resistance in patients with type 2 diabetes mellitus: a preliminary report. Cardiovasc Diabetol. **12**, 21 (2013). 10.1186/1475-2840-12-21 23339473 10.1186/1475-2840-12-21 PMC3608161

